# Supplementary material for: Ligand activation mechanisms of human KCNQ2 channel
Source: Nat Commun. 2023 Oct 19;14:6632. doi: 10.1038/s41467-023-42416-x (PMC10587151; doi:10.1038/s41467-023-42416-x)
Supplement: Supplementary file 1 — Supplementary Information [file 41467_2023_42416_MOESM1_ESM.pdf]

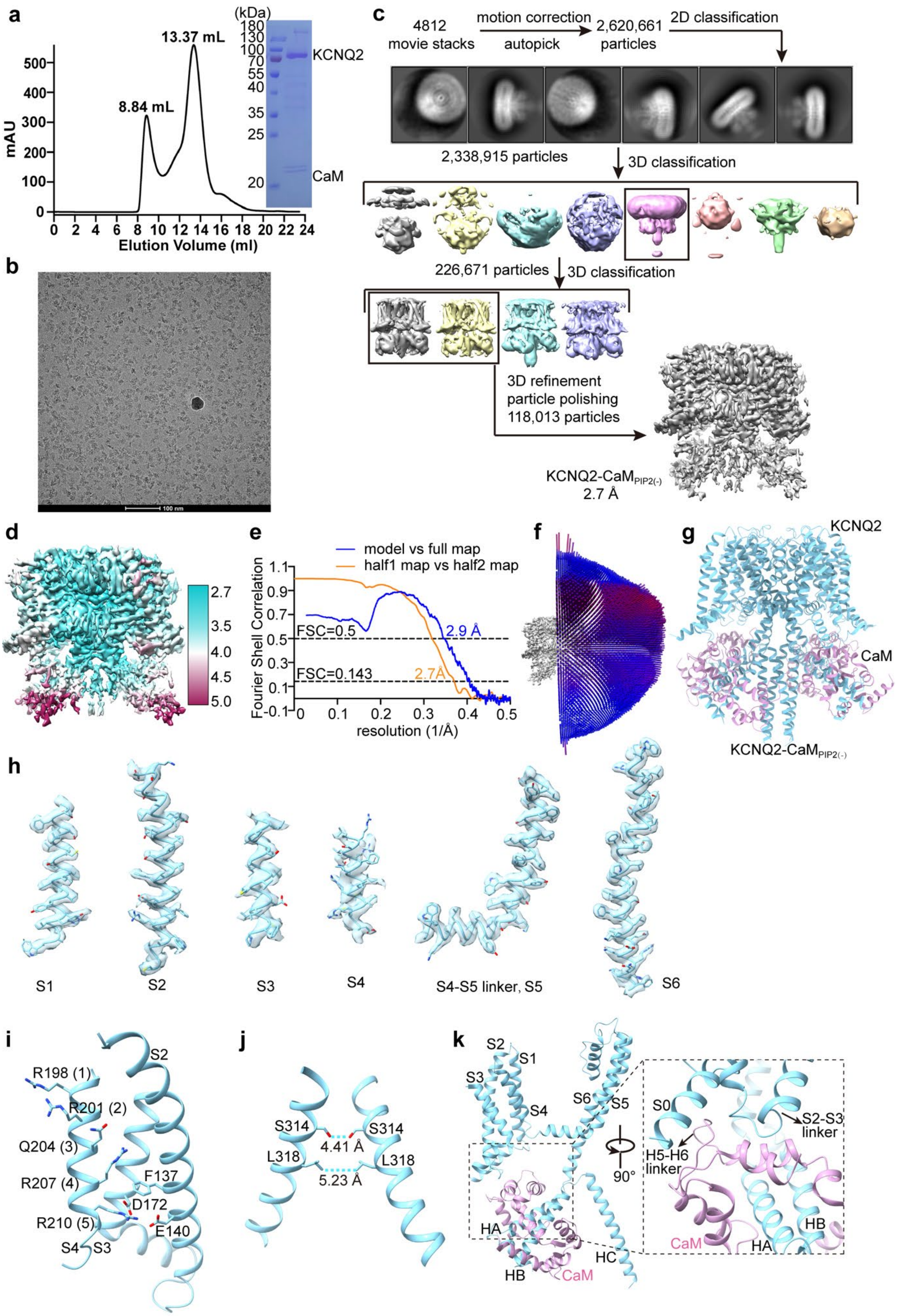

Supplementary Fig. 1 Structure determination of KCNQ2-CaM$_{PIP2(-)}$. a Size-exclusion

chromatography of KCNQ2-CaM on Superose 6 (GE Healthcare) and SDS-PAGE analysis of the final sample for KCNQ2-$CaM_{PIP2(-)}$. The y axis is in mili absorption unit (mAU). Source data are provided in the Source Data file. **b** Representative cryo-EM micrograph of KCNQ2-$CaM_{PIP2(-)}$. **c** Flowchart of image processing for KCNQ2-$CaM_{PIP2(-)}$ particles. **d** The density map of KCNQ2-$CaM_{PIP2(-)}$ colored by local resolution. The local resolution was estimated with RELION 3.1 and generated in Chimera. **e** The Gold-standard Fourier shell correlation (FSC) curves of the final 3D reconstruction of KCNQ2-$CaM_{PIP2(-)}$, and the FSC curve for cross-validation between the map and the model of KCNQ2-$CaM_{PIP2(-)}$. **f** Euler angle distribution of KCNQ2-$CaM_{PIP2(-)}$ particles used in the final 3D reconstruction, with the heights of the cylinders corresponding to the number of particles. **g** The cartoon model of KCNQ2-$CaM_{PIP2(-)}$ in the side view. **h** Sample maps of the KCNQ2-$CaM_{PIP2(-)}$ structure. **i** The activated VSD of KCNQ2-$CaM_{PIP2(-)}$ in the side view with S1 omitted for clarity. **j** The closed activation gate of KCNQ2-$CaM_{PIP2(-)}$. The dashed lines show diagonal atom-to-atom distance (in Å) at the constriction-lining residues Ser314 and Leu318. **k** Interactions between the VSD of KCNQ2 and CaM.

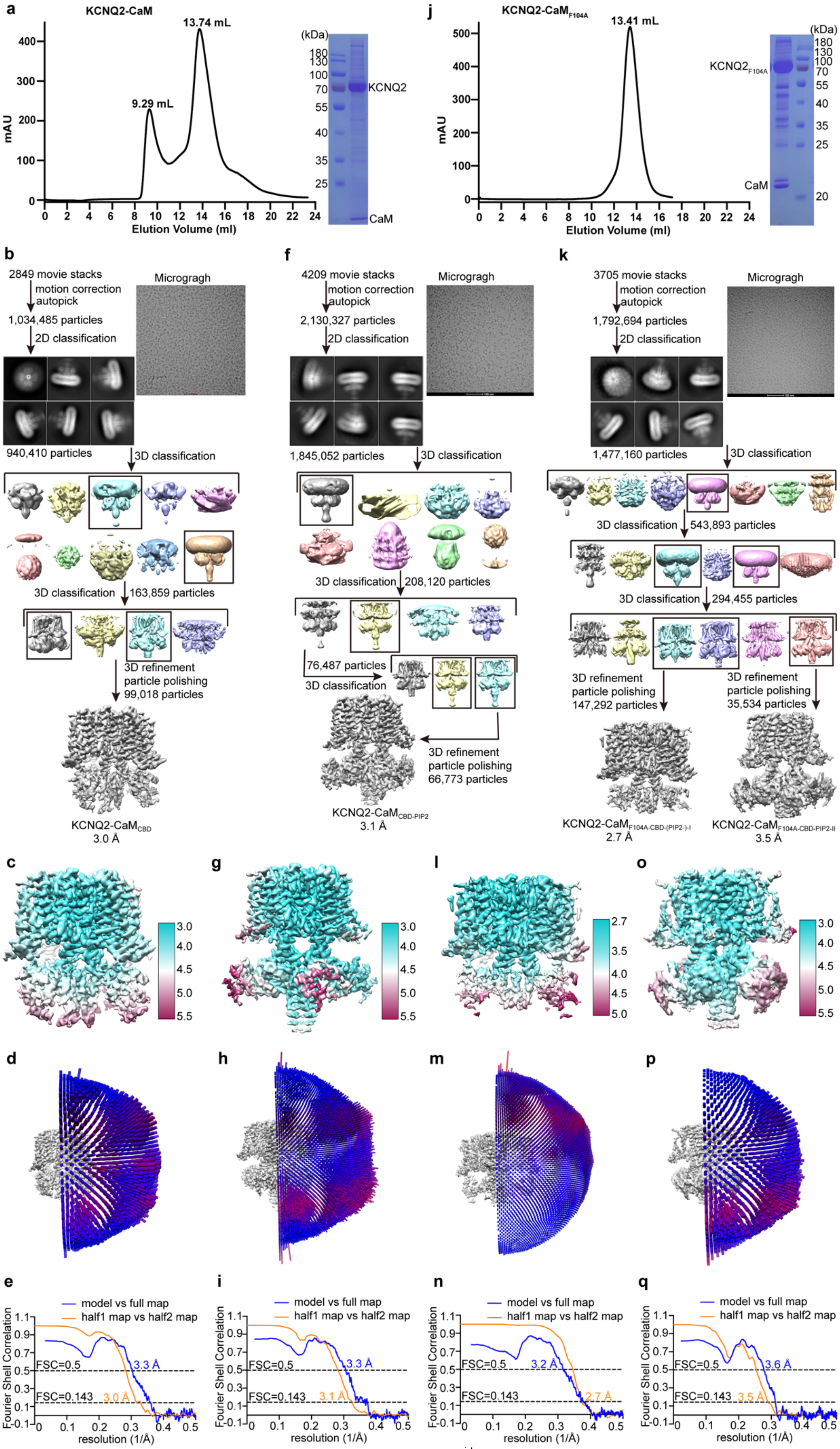
a
KCNQ2-CaM
13.74 mL
9.29 mL
mAU
Elution Volume (ml)
(kDa)
KCNQ2
CaM
j
KCNQ2-CaMF104A
13.41 mL
KCNQ2F104A
b
2849 movie stacks
motion correction
autopick
1,034,485 particles
2D classification
Micrograph
940,410 particles
3D classification
163,859 particles
3D refinement
particle polishing
99,018 particles
KCNQ2-CaMCBD
3.0 Å
f
4209 movie stacks
2,130,327 particles
1,845,052 particles
208,120 particles
76,487 particles
66,773 particles
KCNQ2-CaMCBD-PIP2
3.1 Å
k
3705 movie stacks
1,792,694 particles
1,477,160 particles
543,893 particles
294,455 particles
147,292 particles
35,534 particles
KCNQ2-CaMF104A-CBD-(PIP2)-I
2.7 Å
KCNQ2-CaMF104A-CBD-PIP2-II
3.5 Å
c
g
l
o
d
h
m
p
e
i
n
q
model vs full map
half1 map vs half2 map
Fourier Shell Correlation
resolution (1/Å)
FSC=0.5
FSC=0.143
3.3 Å
3.0 Å
3.1 Å
3.2 Å
2.7 Å
3.6 Å
3.5 Å

Supplementary Fig. 2 Structure determination of KCNQ2-CaM$_{\mathrm{CBD}}$, KCNQ2-CaM$_{\mathrm{CBD\text{-}PIP2}}$, KCNQ2-CaM$_{\mathrm{F104A\text{-}CBD\text{-}PIP2(\text{-})\text{-}I}}$, and CaM$_{\mathrm{F104A\text{-}CBD\text{-}PIP2\text{-}II}}$. a Size-exclusion chromatography of KCNQ2-CaM on Superose 6 (GE Healthcare) and SDS-PAGE analysis of the final sample for KCNQ2-CaM$_{\mathrm{CBD}}$ and KCNQ2-CaM$_{\mathrm{PIP2\text{-}CBD}}$. The y axis is in mili absorption unit (mAU). **b, f, k** Flowchart of image processing for KCNQ2-CaM$_{\mathrm{CBD}}$, KCNQ2-CaM$_{\mathrm{CBD\text{-}PIP2}}$, and KCNQ2-CaM$_{\mathrm{F104A\text{-}CBD\text{-}PIP2}}$ particles, respectively. **c, g, l, o** The density map of KCNQ2-CaM$_{\mathrm{CBD}}$, KCNQ2-CaM$_{\mathrm{CBD\text{-}PIP2}}$, KCNQ2-CaM$_{\mathrm{F104A\text{-}CBD\text{-}(PIP2\text{-})\text{-}I}}$, and KCNQ2-CaM$_{\mathrm{F104A\text{-}CBD\text{-}PIP2\text{-}II}}$ colored by local resolution. The local resolution was estimated with RELION 3.1 and generated in Chimera. **d, h, m, p** Euler angle distribution of particles included in the final *C4*-symmetric 3D reconstruction of KCNQ2-CaM$_{\mathrm{CBD}}$, KCNQ2-CaM$_{\mathrm{CBD\text{-}PIP2}}$, KCNQ2-CaM$_{\mathrm{F104A\text{-}CBD\text{-}(PIP2\text{-})\text{-}I}}$, and KCNQ2-CaM$_{\mathrm{F104A\text{-}CBD\text{-}PIP2\text{-}II}}$, respectively. **e, i, n, q** The Gold-standard Fourier shell correlation (FSC) curves of the final 3D reconstruction of KCNQ2-CaM$_{\mathrm{CBD}}$, KCNQ2-CaM$_{\mathrm{CBD\text{-}PIP2}}$, KCNQ2-CaM$_{\mathrm{F104A\text{-}CBD\text{-}(PIP2\text{-})\text{-}I}}$, and KCNQ2-CaM$_{\mathrm{F104A\text{-}CBD\text{-}PIP2\text{-}II}}$, and the FSC curve for cross-validation between the map and the model of KCNQ2-CaM$_{\mathrm{CBD}}$, KCNQ2-CaM$_{\mathrm{CBD\text{-}PIP2}}$, KCNQ2-CaM$_{\mathrm{F104A\text{-}CBD\text{-}(PIP2\text{-})\text{-}I}}$, and KCNQ2-CaM$_{\mathrm{F104A\text{-}CBD\text{-}PIP2\text{-}II}}$, respectively. **j** Size-exclusion chromatography of KCNQ2-CaM$_{\mathrm{F104A}}$ on Superose 6 (GE Healthcare) and SDS-PAGE analysis of the final sample for KCNQ2-CaM$_{\mathrm{F104A\text{-}CBD\text{-}PIP2}}$. The y axis is in mili absorption unit (mAU). For **a** and **j,** source data are provided in the Source Data file.

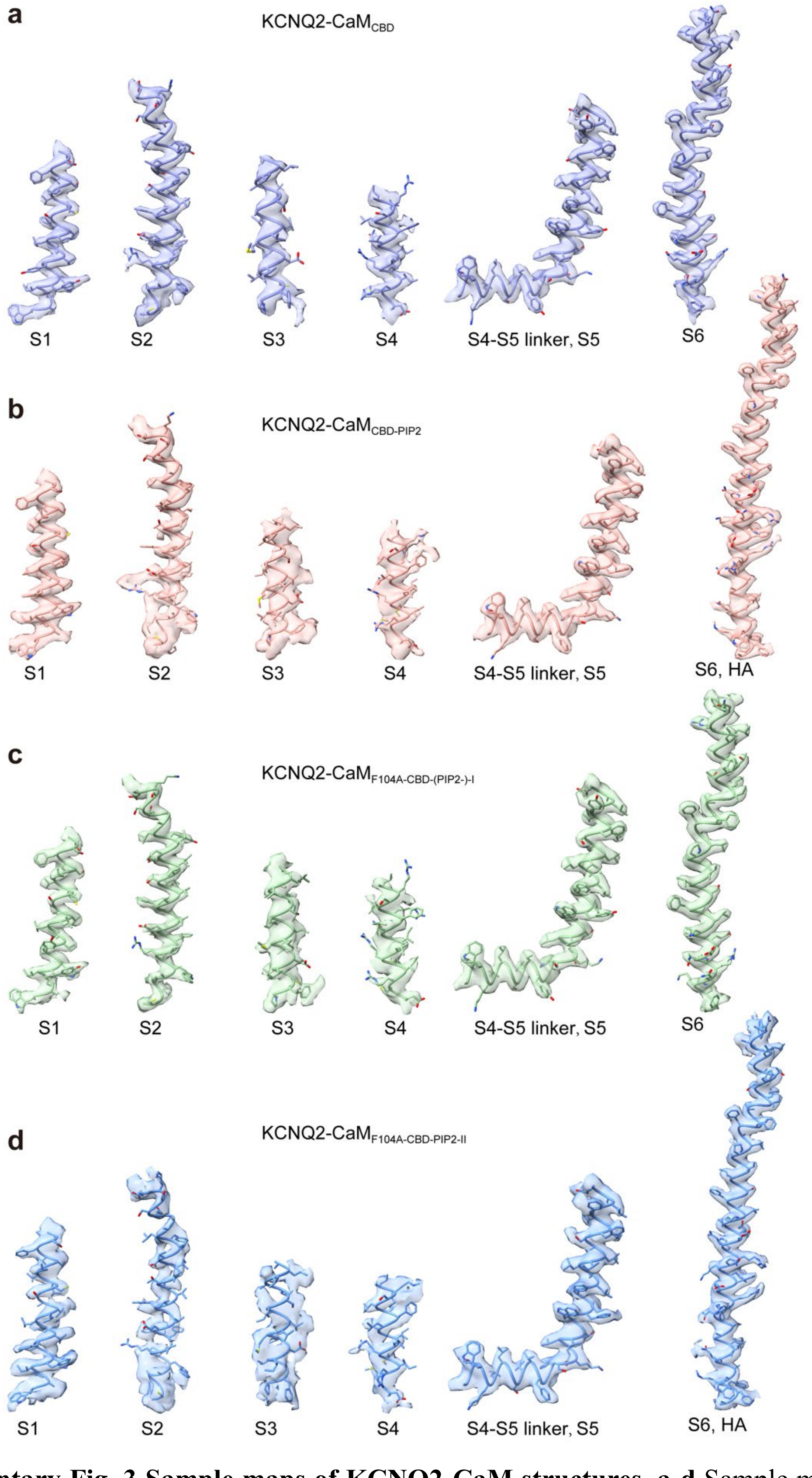

Supplementary Fig. 3 Sample maps of KCNQ2-CaM structures. a-d Sample maps of the KCNQ2-CaM$_{\text{CBD}}$, KCNQ2-CaM$_{\text{CBD-PIP2}}$, KCNQ2-CaM$_{\text{F104A-CBD-PIP2(-)-I}}$, and KCNQ2-CaM$_{\text{F104A-CBD-PIP2(-)-II}}$ structures.

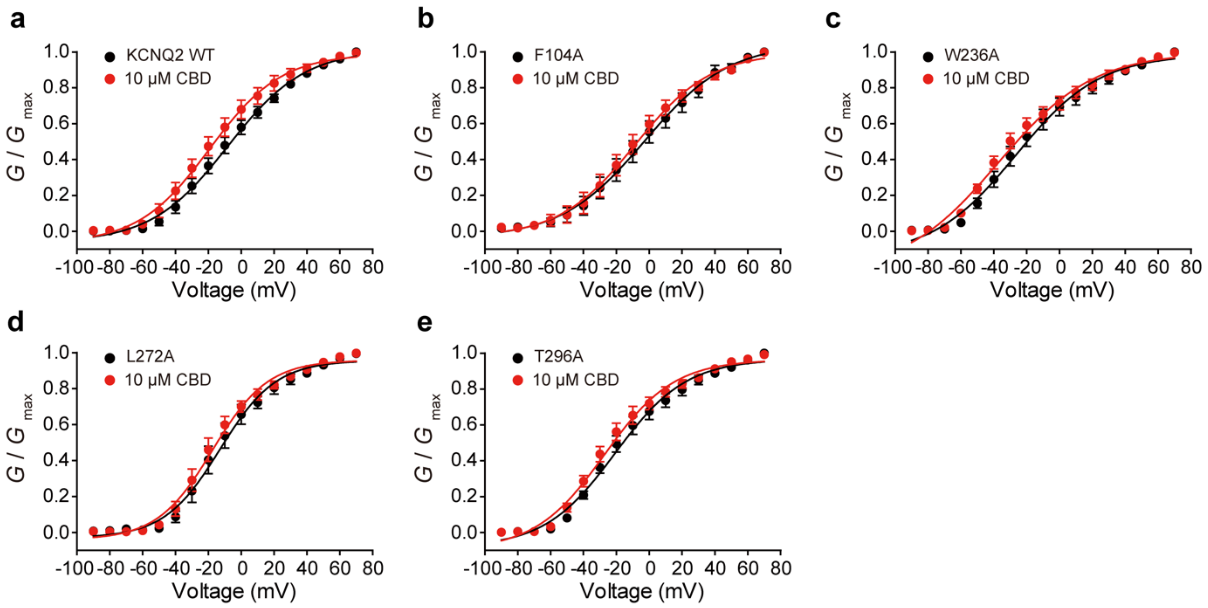

Supplementary Fig. 4 Voltage-dependent activation curves of WT and mutant KCNQ2 channels before (black) and after (red) application of 10 μM CBD. a-e Normalized *G-V* relations of WT KCNQ2, F104A, W236A, L272A, and T296A mutants. CHO cells transiently expressing the channels were held at -80 mV and depolarizing voltage steps from -90 mV to +70 mV with 10 mV increments were applied for 1500 ms, followed by a hyperpolarizing step to -120 mV for 500 ms. The conductance was normalized to the maximal conductance of each treatment (G / G_{max}) and the voltage-dependent activation curve fitted with the Boltzmann equation. Data are presented as mean ± SEM. For W236A, n = 3 individual cells; for WT and all the other mutants, n = 5 individual cells. Source data are included in the Source Data file.

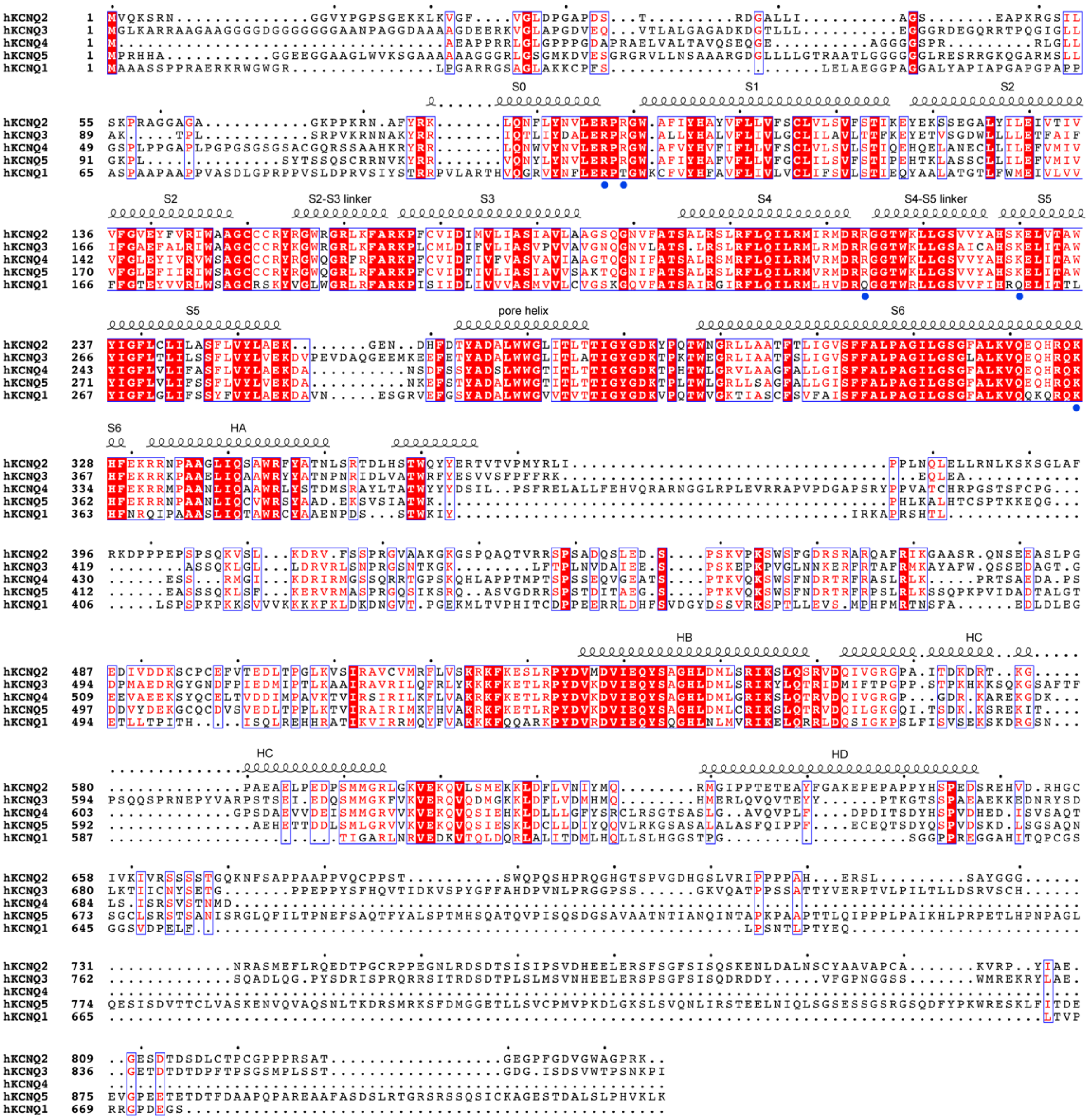

Supplementary Fig. 5 Sequence alignment of KCNQ1-5. Secondary structures are assigned based on the structure of KCNQ2-CaM$_{apo}$ (PDB: 7CR3). The PIP_2-interacting residues are marked with blue dots.

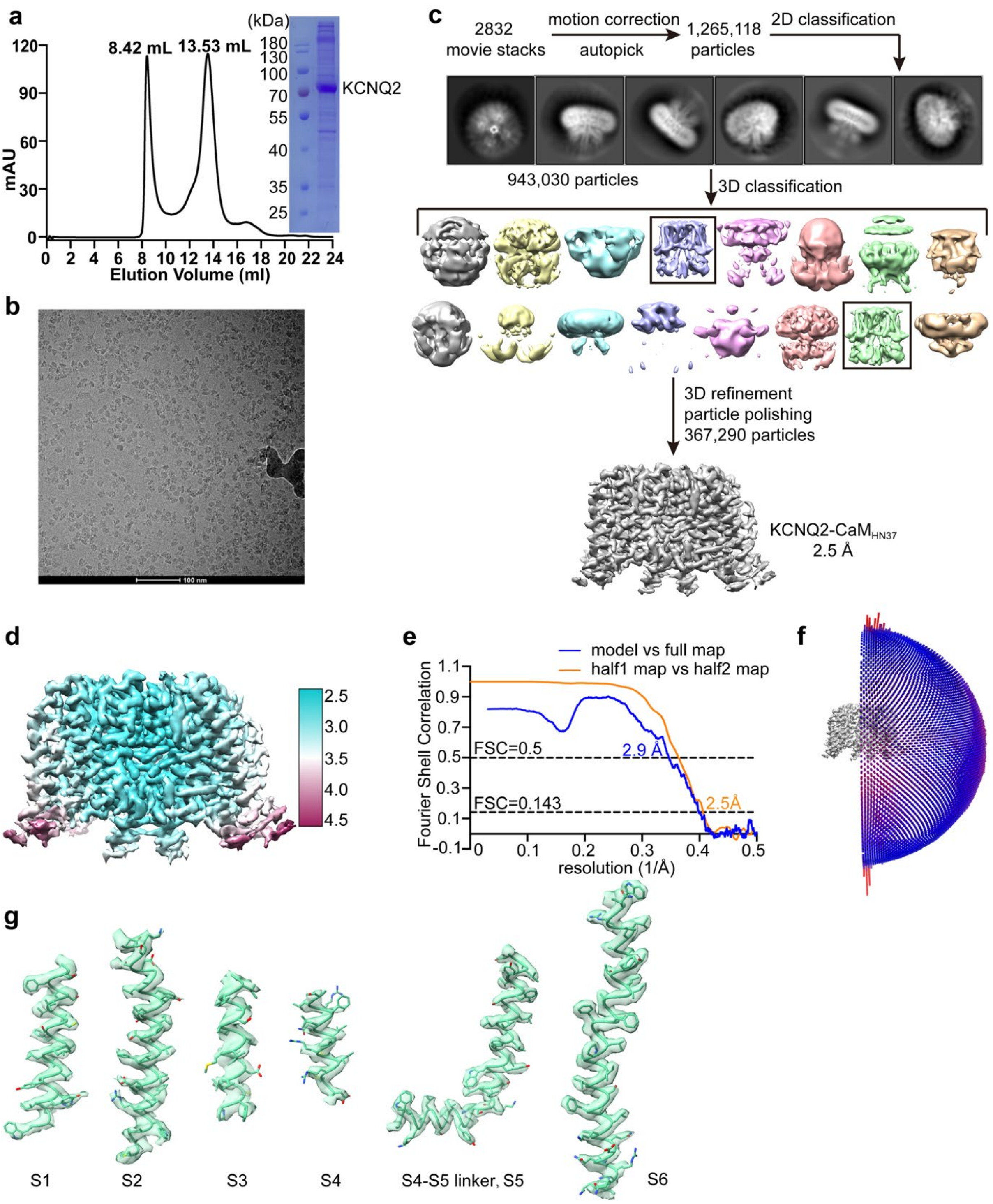

Supplementary Fig. 6 Structure determination of KCNQ2-CaM$_{HN37}$. a Size-exclusion chromatography of KCNQ2-CaM on Superose 6 (GE Healthcare) and SDS-PAGE analysis of the final sample for KCNQ2-CaM$_{HN37}$. The y axis is in mili absorption unit (mAU). Source data are included in the Source Data file. **b** Representative cryo-EM micrograph of KCNQ2-CaM$_{HN37}$. **c** Flowchart of image processing for KCNQ2-CaM$_{HN37}$ particles. **d** The density map

of KCNQ2-CaM$_{HN37}$ colored by local resolution. The local resolution was estimated with RELION 3.1 and generated in Chimera. **e** The Gold-standard Fourier shell correlation (FSC) curves of the final 3D reconstruction of KCNQ2-CaM$_{HN37}$, and the FSC curve for cross-validation between the map and the model of KCNQ2-CaM$_{HN37}$. **f** Euler angle distribution of KCNQ2-CaM$_{HN37}$ particles used in the final 3D reconstruction, with the heights of the cylinders corresponding to the number of particles. **g** Sample maps of the KCNQ2-CaM$_{HN37}$ structure.

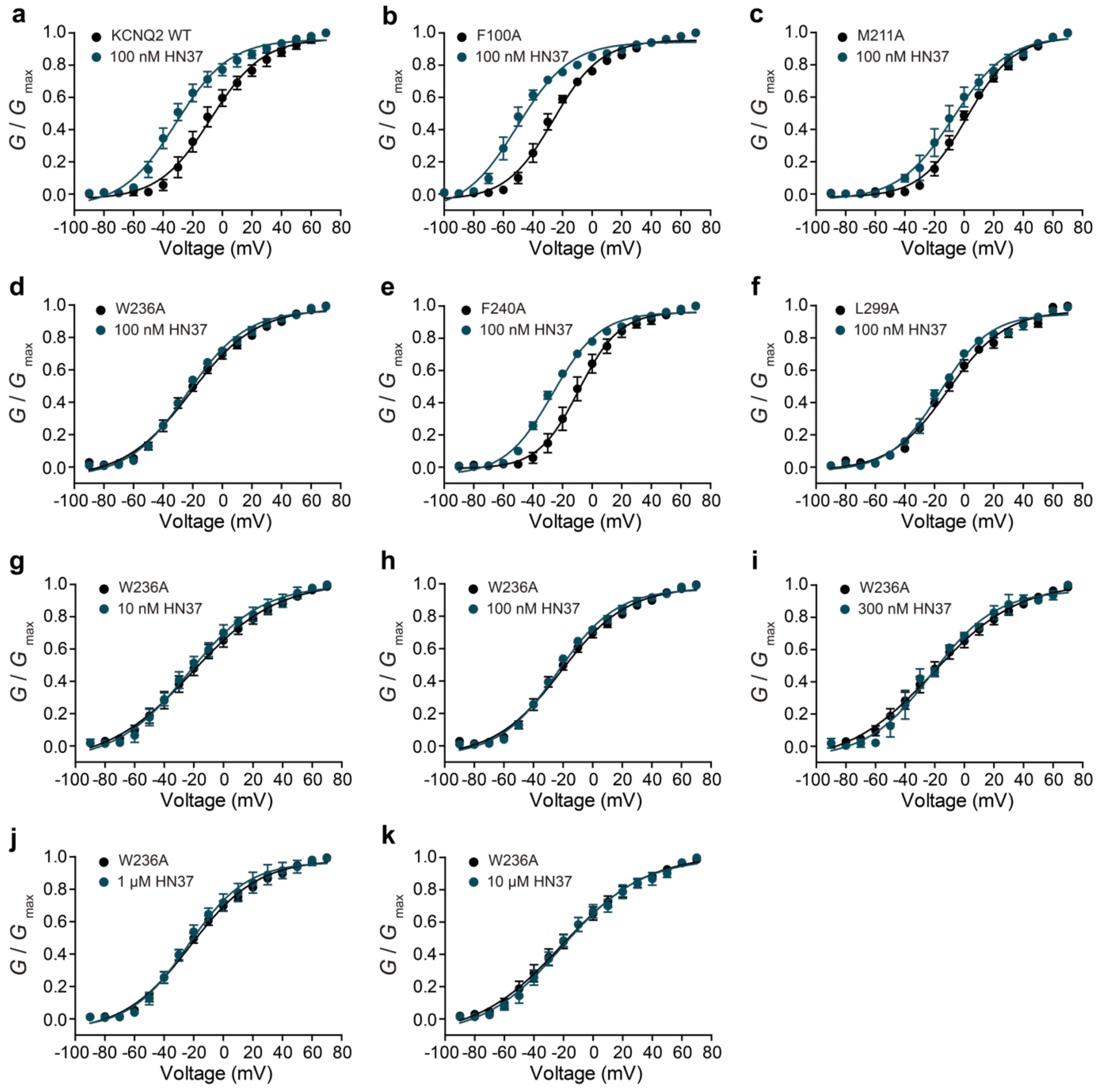

Supplementary Fig. 7 Voltage-dependent activation curves of WT and mutant KCNQ2 channels before (black) and after (blue) application of 100 nM HN37. a-f Normalized *G-V* relations of WT KCNQ2 (n = 5), F100A (n = 4), M211A (n = 4), W236A (n = 4), F240A (n = 5), and L299A (n = 3) mutants. CHO cells transiently expressing the channels were held at -100 mV and depolarizing voltage steps from –100 mV or -90 mV to +70 mV with 10 mV increments were applied for 1500 ms, followed by a hyperpolarizing step to -120 mV for 500 ms. The conductance was normalized to the maximal conductance of each treatment (G / G_{max}) and the voltage-dependent activation curve fitted with the Boltzmann equation. Data are presented as means ± SEM. n indicates the number of experiments from individual cells. **g-k**

The sensitivity of the KCNQ2 W236A mutant to HN37. CHO cells transiently expressing W236A mutant were held at -100 mV and depolarizing voltage steps from -90 mV to +70 mV with 10 mV increments were applied for 1500 ms, followed by a hyperpolarizing step to -120 mV for 500 ms. The conductance values before (black) and after (red) application of HN37 at indicated concentration were separately normalized to the maximal conductance of each treatment (G / G_{max}) and then the voltage-dependent activation curve fitted with the Boltzmann equation. Data are presented as means ± SEM. n values are 5 for 10 nM HN37, 6 for 100 nM HN37, 3 for 300 nM HN37, 6 for 1 μM HN37, and 5 for 10 μM HN37. n indicates the number of experiments from individual cells. Source data are included as a Source Data file.

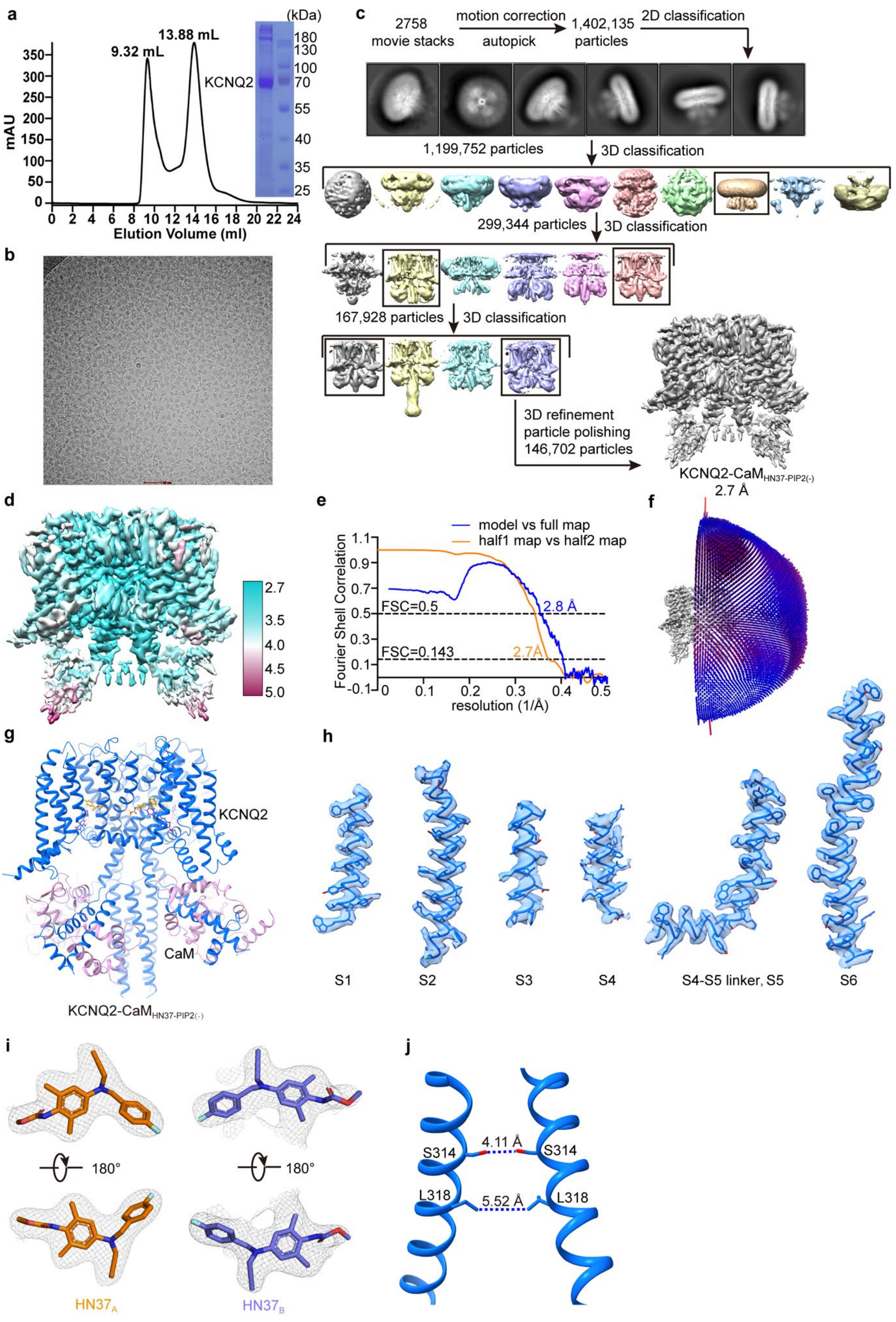

Supplementary Fig. 8 Structure determination of KCNQ2-CaM$_{\mathrm{HN37\text{-}PIP2(-)}}$. a Size-

exclusion chromatography of KCNQ2-CaM on Superose 6 (GE Healthcare) and SDS-PAGE analysis of the final sample for KCNQ2-CaM$_{HN37\text{-}PIP2(-)}$. The y axis is in mili absorption unit (mAU). Source data are included in the Source Data file. **b** Representative cryo-EM micrograph of KCNQ2-CaM$_{HN37\text{-}PIP2(-)}$. **c** Flowchart of image processing for KCNQ2-CaM$_{HN37\text{-}PIP2(-)}$ particles. **d** The density map of KCNQ2-CaM$_{HN37\text{-}PIP2(-)}$ colored by local resolution. The local resolution was estimated with RELION 3.1 and generated in Chimera. **e** The Gold-standard Fourier shell correlation (FSC) curves of the final 3D reconstruction of KCNQ2-CaM$_{HN37\text{-}PIP2(-)}$, and the FSC curve for cross-validation between the map and the model of KCNQ2-CaM$_{HN37\text{-}PIP2(-)}$. **f** Euler angle distribution of KCNQ2-CaM$_{HN37\text{-}PIP2(-)}$ particles used in the final 3D reconstruction, with the heights of the cylinders corresponding to the number of particles. **g** The cartoon model of KCNQ2-CaM$_{HN37\text{-}PIP2(-)}$ in the side view. **h** Sample maps of the KCNQ2-CaM$_{HN37\text{-}PIP2(-)}$ structure. **i** The density maps of two HN37 molecules in different orientations at the contour level of 4.5 σ. **j** The closed activation gate of KCNQ2-CaM$_{HN37\text{-}PIP2(-)}$. The dashed lines show diagonal atom-to-atom distance (in Å) at the constriction-lining residues Ser314 and Leu318.

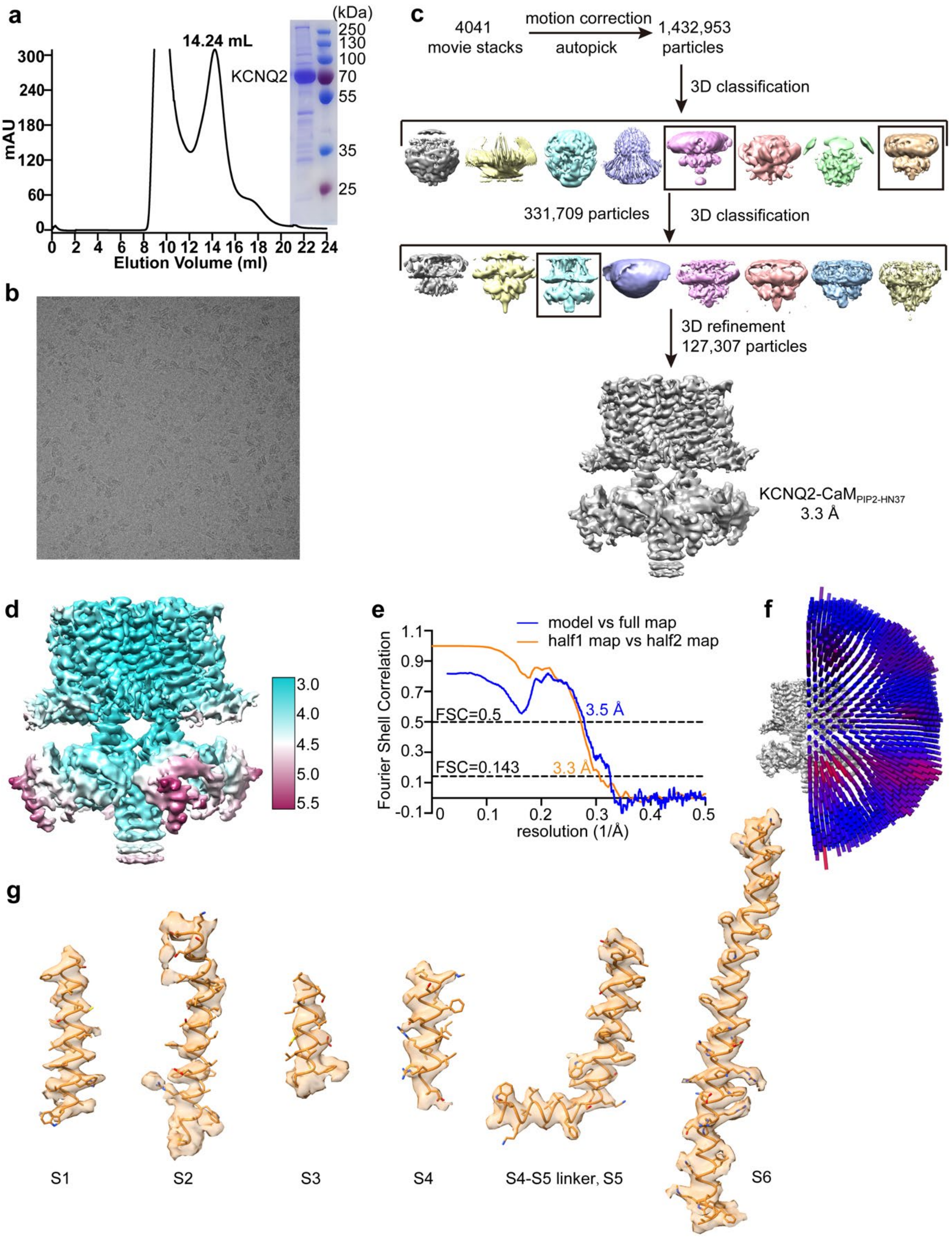

Supplementary Fig. 9 Structure determination of KCNQ2-CaM$_{\text{PIP2-HN37}}$. a Size-exclusion chromatography of KCNQ2-CaM on Superose 6 (GE Healthcare) and SDS-PAGE analysis of the final sample for KCNQ2-CaM $_{\text{PIP2-HN37}}$. The y axis is in mili absorption unit (mAU). Source data are included in the Source Data file. **b** Representative cryo-EM micrograph of KCNQ2-

$CaM_{PIP2\text{-}HN37}$. **c** Flowchart of image processing for KCNQ2-$CaM_{PIP2\text{-}HN37}$ particles. **d** The density map of KCNQ2-$CaM_{PIP2\text{-}HN37}$ colored by local resolution. The local resolution was estimated with RELION 3.1 and generated in Chimera. **e** The Gold-standard Fourier shell correlation (FSC) curves of the final 3D reconstruction of KCNQ2-$CaM_{PIP2\text{-}HN37}$, and the FSC curve for cross-validation between the map and the model of KCNQ2-$CaM_{PIP2\text{-}HN37}$. **f** Euler angle distribution of KCNQ2-$CaM_{PIP2\text{-}HN37}$ particles used in the final 3D reconstruction, with the heights of the cylinders corresponding to the number of particles. **g** Sample maps of the KCNQ2-$CaM_{PIP2\text{-}N37}$ structure.

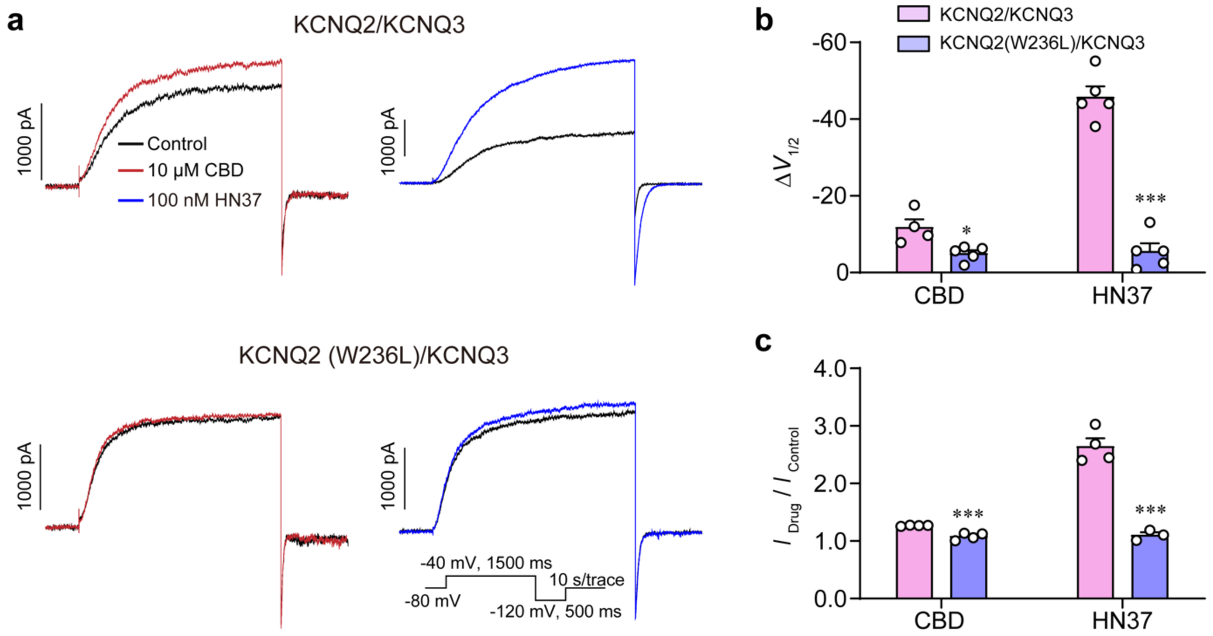

Supplementary Fig. 10 The activation effects of CBD and HN37 on the KCNQ2/KCNQ3 and KCNQ2(W236L)/KCNQ3 hetero-tetramers. **a** Representative current traces of KCNQ2/KCNQ3 and KCNQ2(W236L)/KCNQ3 hetero-tetramers before and after application of 10 µM CBD or 100 nM HN37. *Inset,* the recording protocol. **b** The half-maximal activation voltage shift *($\Delta V_{1/2}$)* for CBD or HN37 on the hetero-tetramers calculated from the panel **a**. Data are presented as mean ± SEM. An unpaired two-tailed t-test was used to make a comparison between the KCNQ2/KCNQ3 and KCNQ2(W236L)/KCNQ3. *p* and n values are **p* = 0.0143 and n = 4 or 5 for CBD, ****p* = 0.0001 and n = 5 for HN37. n represents the number of experiments from individual cells. **c** The activation efficacy (I_{Drug} / $I_{Control}$) for CBD or HN37 on the hetero-tetramers calculated from the panel a. Data are presented as mean ± SEM. An unpaired two-tailed t-test was used to make a comparison between the KCNQ2/KCNQ3 and KCNQ2(W236L)/KCNQ3. *p* and n values are ****p* = 0.0009 and n = 4 for CBD, ****p* = 0.0003 and n = 3 or 4 for HN37. n indicates the number of experiments from individual cells. Source data are included in the Source Data file.

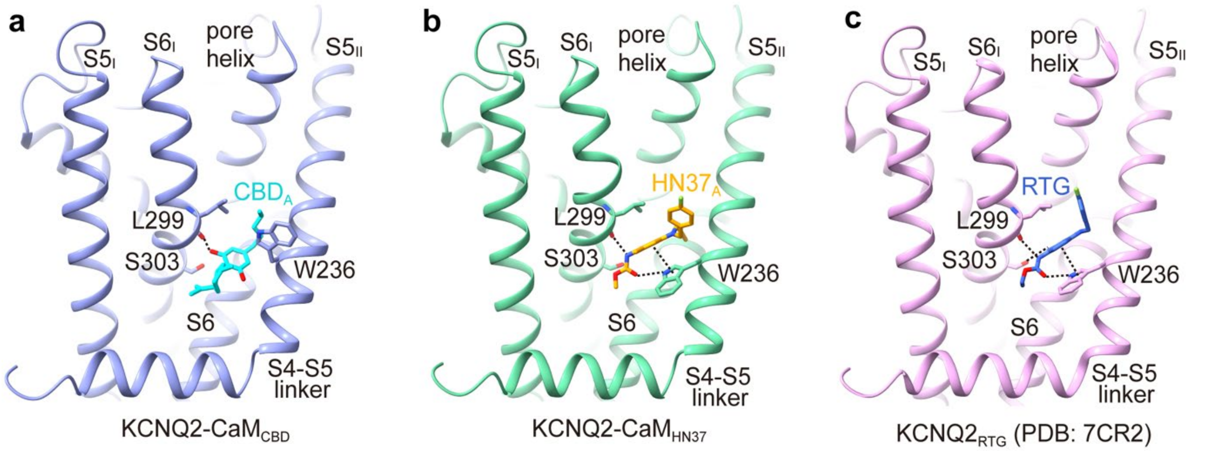

Supplementary Fig. 11 Comparison of ligand binding sites in KCNQ2. a The CBD_A binding site in $KCNQ2\text{-}CaM_{CBD}$. The side chains of Trp236, Lue299, and Ser303 are shown as sticks. **b** The $HN37_A$ binding site in $KCNQ2\text{-}CaM_{HN37}$. The side chains of Trp236, Lue299, and Ser303 are shown as sticks. **c** The RTG binding site in $KCNQ2_{RTG}$ (PDB:7CR2). The side chains of Trp236, Lue299, and Ser303 are shown as sticks.

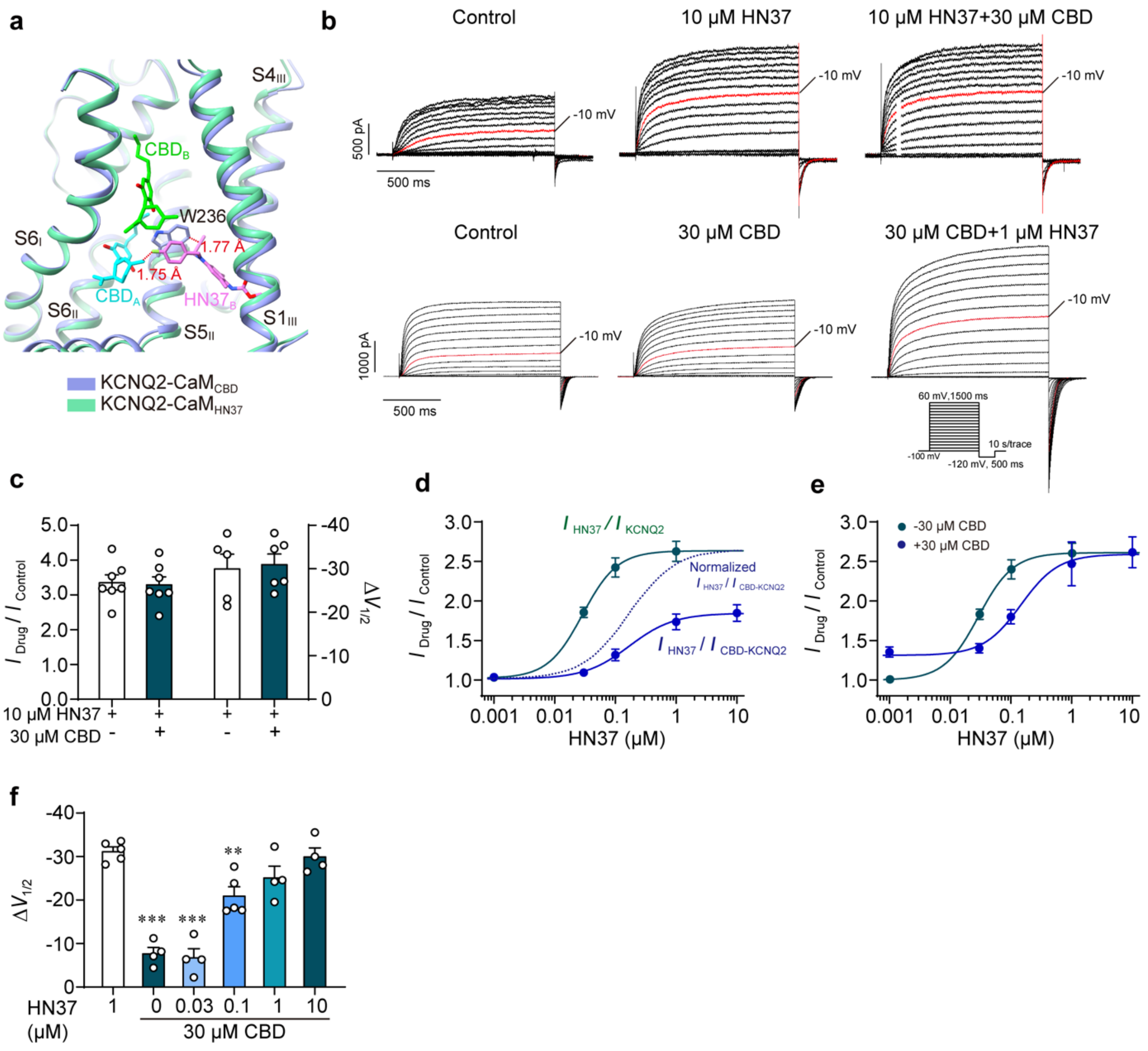

Supplementary Fig. 12 The competitive binding of HN37 and CBD. **a** Structural alignment of KCNQ2-CaM$_{CBD}$ and KCNQ2-CaM$_{HN37}$. **b** Representative current traces of KCNQ2 channels after sequential perfusion of 10 μM HN37 and 30 μM CBD + 10 μM HN37 (up), 30 μM CBD and 30 μM CBD + 1 μM HN37 (down). **c** The activation efficacy (I_{Drug} / $I_{Control}$) and half maximal activation voltage shift ($\Delta V_{1/2}$) for 10 μM HN37 on KCNQ2 channels with or without 30 μM CBD. Dara are presented as mean ± SEM. An unpaired two-tailed t-test was used to make a comparison between the 10 μM HN37 and 10 μM HN37 + 30 μM CBD. For $I_{drug} / I_{control}$, $p = 0.8433$ for 10 μM HN37 + 30 μM CBD (n = 7) compared to 10 μM HN37 (n =7); for $\Delta V_{1/2}$, $p = 0.8074$ for 10 μM HN37 + 30 μM CBD (n = 6) compared to 10 μM HN37 (n = 5). n indicates the number of experiments from individual cells. **d** Dose-response curves of the activation efficacy measured at -10 mV for HN37 on KCNQ2 or CBD-bound KCNQ2

channel. The maximal activation efficacy of HN37 on CBD-bound KCNQ2 channel (I_{HN37} / $I_{CBD-KCNQ2}$) dramatically decreased in the comparison of native KCNQ2 channel (I_{HN37} / I_{KCNQ2}). Dara are presented as mean ± SEM. n = 3-8 for I_{HN37} / $I_{CBD-KCNQ2}$ and 5-8 for I_{HN37} / I_{KCNQ2}. n indicates the number of experiments from individual cells. **e** Dose-response curves of the activation efficacy for HN37 on KCNQ2 in the absence and presence of 30 μM CBD. Dara are presented as mean ± SEM. For in the absence of 30 μM CBD, n = 5-8; for in the presence of 30 μM CBD, n = 3-8. n indicates the number of experiments from individual cells. **f** The $\Delta V_{1/2}$ for HN37 on KCNQ2 channels with or without 30 μM CBD. Dara are presented as mean ± SEM. One-way ANOVA with Dunnett's multiple comparisons test was applied. *p* and n values compared to 1 μM HN37 (n = 5) were ***p = 0.0001 and n = 4 for 30 μM CBD, ***p = 0.0001 and n = 4 for 0.03 μM HN37 + 30 μM CBD, **p = 0.0025 and n = 5 for 0.1 μM HN37 + 30 μM CBD, p = 0.1246 and n = 4 for 1 μM HN37 + 30 μM CBD, and p = 0.9897 and n = 4 for 10 μM HN37 + 30 μM CBD. n indicates the number of experiments from individual cells. For **c-f**, source data are included in the Source Data file.

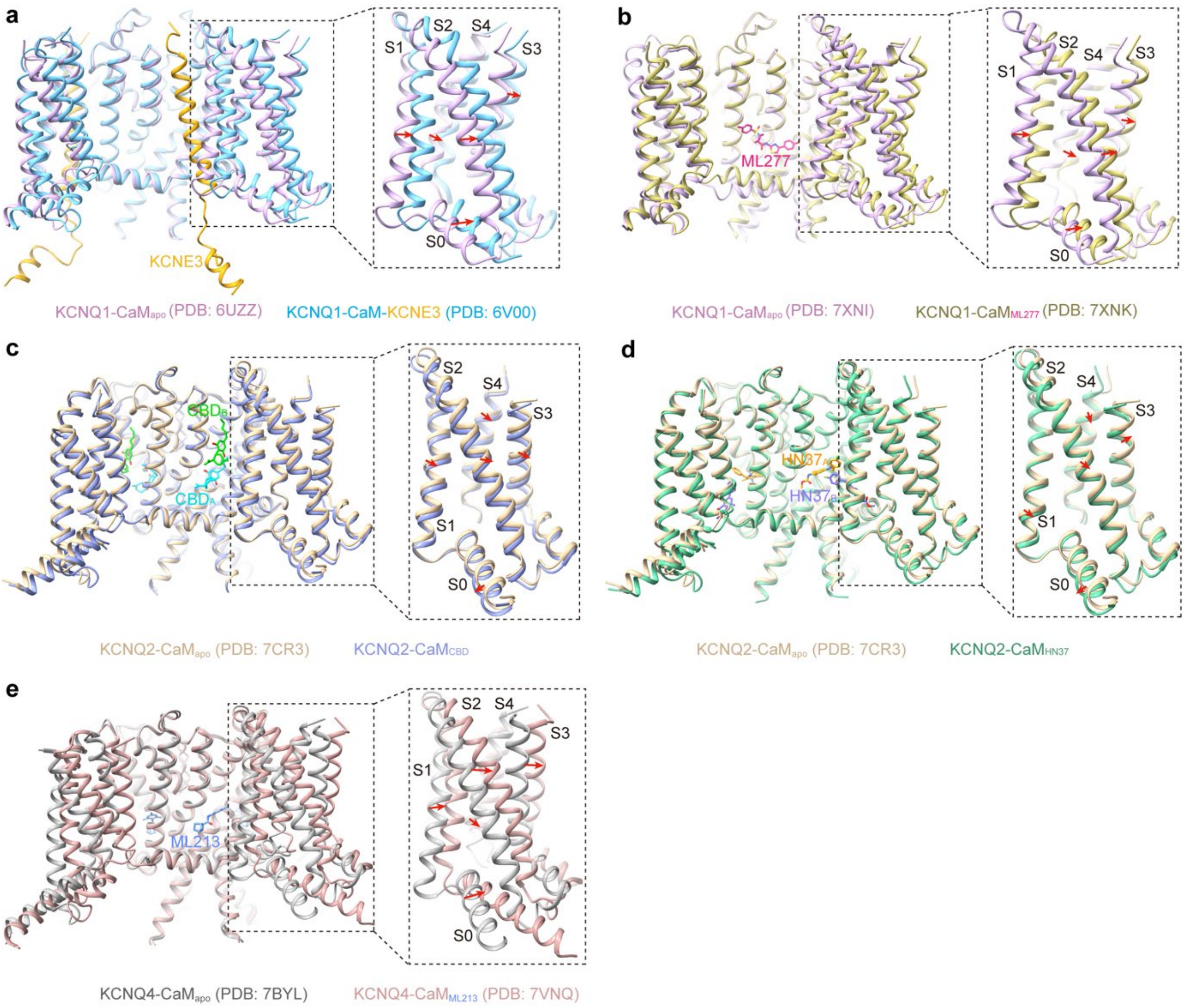

Supplementary Fig. 13 Analyses of the modulation of KCNQ channels by KCNE and ligands. a Structural comparison of KCNQ1-CaM$_{apo}$ (PDB: 6UZZ) and KCNQ1-CaM-KCNE3 (PDB: 6V00). Red arrows indicate the shift of VSD upon the KCNE3 binding. **b** Structural comparison of KCNQ1-CaM$_{apo}$ (PDB: 7XNI) and KCNQ1-CaM$_{ML277}$ (PDB: 7XNK). Red arrows indicate the shift of VSD upon the ML277 binding. **c** Structural comparison of KCNQ2-CaM$_{apo}$ (PDB:7CR3) and KCNQ2-CaM$_{CBD}$. Red arrows indicate the shift of VSD upon the CBD binding. **d** Structural comparison of KCNQ2-CaM$_{apo}$ (PDB: 7CR3) and KCNQ2-CaM$_{HN37}$. Red arrows indicate the shift of VSD upon the HN37 binding. **e** Structural comparison of KCNQ4-CaM$_{apo}$ (PDB: 7BYL) and KCNQ4-CaM$_{ML213}$. Red arrows indicate the shift of VSD upon the ML213 binding.

Supplementary Table 1. Data collection and refinement statistics.

	KCNQ2-CaM$_{PIP2(-)}$ (EMD-35884) (PDB 8J05)	KCNQ2-CaM$_{CBD}$ (EMD-35879) (PDB 8J00)	KCNQ2-CaM$_{CBD-PIP2}$ (EMD-35880) (PDB 8J01)	KCNQ2-CaM$_{F104A-CBD-PIP2(-)-I}$ (EMD-35882) (PDB 8J03)	KCNQ2-CaM$_{F104A-CBD-PIP2-II}$ (EMD-35881) (PDB 8J02)
**Data collection and processing**					
Magnification	130000	130000	130000	130000	130000
Voltage (kV)	300	300	300	300	300
Electron exposure (e-/Å^2)	~ 52	~ 52	~ 52	~ 52	~ 52
Defocus range (μm)	-0.8 to -1.5	-0.8 to -1.5	-0.8 to -1.5	-0.8 to -1.5	-0.8 to -1.5
Pixel size (Å)	0.93	0.93	0.93	0.93	0.93
Symmetry imposed	*C4*	*C4*	*C4*	*C4*	*C4*
Initial particle images (no.)	2,620,661	1,034,485	2,130,327	1,792,694	1,792,694
Final particle images (no.)	118,013	99,018	66,773	147,292	35,534
Map resolution (Å)	2.7	3.0	3.1	2.7	3.5
FSC threshold	0.143	0.143	0.143	0.143	0.143
**Refinement**					
Initial model used (PDB code)	7CR3	7CR3	7CR3	7CR3	7CR3
Model resolution (Å)	2.9	3.3	3.3	3.2	3.6
FSC threshold	0.5	0.5	0.5	0.5	0.5
Map sharpening B factor (Å^2)	-40	-40	-30	-40	-40
Model composition					
Non-hydrogen atoms	15876	15240	16248	15232	16132
Protein residues	1960	1876	1988	1892	1988
ligands	0	8	12	4	8
B factors (Å^2)					
Protein	154.57	164.94	115.94	182.16	140.46
R.m.s. deviations					
Bond lengths (Å)	0.003	0.003	0.003	0.003	0.003
Bond angles (°)	0.469	0.559	0.548	0.522	0.579
Validation					
MolProbity score	1.71	1.67	1.71	2.16	2.01
Clashscore	8.75	9.81	10.36	11.83	15.74
Rotamer outliers (%)	0.72	0.00	0.60	1.39	0.48
Ramachandran plot					
Favored (%)	96.37	97.13	96.98	95.65	95.50
Allowed (%)	3.63	2.87	3.02	4.35	4.50
Outliers (%)	0.00	0.00	0.00	0.00	0.00

Supplementary Table 1. Data collection and refinement statistics (continued).

	KCNQ2-CaM$_{HN37}$ (EMD-35877) (PDB 8IZY)	KCNQ2-CaM$_{HN37-PIP2(-)}$ (EMD-35883) (PDB 8J04)	KCNQ2-CaM$_{PIP2-HN37}$ (EMD-37270) (PDB 8W4U)
**Data collection and processing**			
Magnification	130000	130000	130000
Voltage (kV)	300	300	300
Electron exposure (e-/Å^2)	~ 64	~ 52	~ 52
Defocus range (μm)	-1.1 to -1.3	-0.8 to -1.5	-0.8 to -1.5
Pixel size (Å)	1.014	0.93	0.93
Symmetry imposed	*C4*	*C4*	*C4*
Initial particle images (no.)	1,265,118	1,402,135	1,432,953
Final particle images (no.)	367,290	146,702	127,307
Map resolution (Å)	2.5	2.7	3.3
FSC threshold	0.143	0.143	0.143
**Refinement**			
Initial model used (PDB code)	7CR3	7CR3	7CR3
Model resolution (Å)	2.9	2.8	3.5
FSC threshold	0.5	0.5	0.5
Map sharpening B factor (Å^2)	-70	-30	-75
Model composition			
Non-hydrogen atoms	8416	15580	16148
Protein residues	1004	1900	1988
ligands	8	8	8
B factors (Å^2)			
Protein	81.26	166.13	148.83
R.m.s. deviations			
Bond lengths (Å)	0.003	0.003	0.003
Bond angles (°)	0.529	0.507	0.590
Validation			
MolProbity score	1.61	1.72	1.83
Clashscore	7.39	8.92	14.12
Rotamer outliers (%)	0.71	0.99	0.54
Ramachandran plot			
Favored (%)	96.76	96.41	97.03
Allowed (%)	3.24	3.59	2.97
Outliers (%)	0.00	0.00	0.00
