# Supplementary material for: Ligand activation mechanisms of human KCNQ2 channel
Source: Nat Commun. 2023 Oct 19;14:6632. doi: 10.1038/s41467-023-42416-x (PMC10587151; doi:10.1038/s41467-023-42416-x)
Supplement: Supplementary file 3 — Description of Additional Supplementary Files [file 41467_2023_42416_MOESM3_ESM.pdf]

Description of Additional Supplementary Files

File Name: Supplementary Data 1
Description: Sequences of primers for the mutagenesis study of KCNQ2.

File Name: Supplementary Movie 1
Description: PIP_2-induced structural rearrangement of CTD and CaM in the context of the whole channel complex.

File Name: Supplementary Movie 2
Description: PIP_2-induced structural rearrangement of CTD and CaM in one KCNQ2 subunit.

File Name: Supplementary Movie 3
Description: PIP_2-induced opening of the activation gate of KCNQ2 in the bottom view.
